# Active Contraction in the Stable Mechanical Environment of the Tunic of the Ascidian, *Halocynthia roretzi,* a Polysaccharide-Based Tissue with Blood Circulatory System

**DOI:** 10.3390/polym15214329

**Published:** 2023-11-05

**Authors:** Yoko Kato

**Affiliations:** Faculty of Engineering, Tohoku Gakuin University, Sendai 984-8588, Japan; ykato@mail.tohoku-gakuin.ac.jp; Tel.: +81-22-354-8731

**Keywords:** cellulose, sulfated chitin, *Halocynthia roretzi*, tunic, active deformation, mechanical environment, finite element method, blood circulation, compressibility

## Abstract

*Halocynthia roretzi*, a member of Ascidiacea, is covered with its own tunic, which is composed of polysaccharides, such as cellulose Iβ and sulfated chitin. *H. roretzi* has an open-vessel system, whose blood vessels and hemocytes are found in the tunic, so that the mechanical environment of the tunic could be carefully controlled because of its influence on hemocyte behaviors. While active deformation of the tunic and related phenomena have been previously reported, the mechanical environment in the tunic, which directly influences its deformation, has been rarely investigated. Meanwhile, the developments of actuators based on cellulose and chitin have been frequently reported. However, a cellulose–sulfated chitin actuator has not been proposed. In this study, the mechanical environment of the tunic, which has been rarely investigated despite its importance in the active deformation of the tunic, was evaluated using finite element analysis. A finite element model of the tunic, based on its histological characteristics as well as deformation patterns, was developed. The results showed that the shape of the tunic, the pattern of fiber distribution, and control of the water content influenced the mechanical environment.

## 1. Introduction

**Figure 1:**
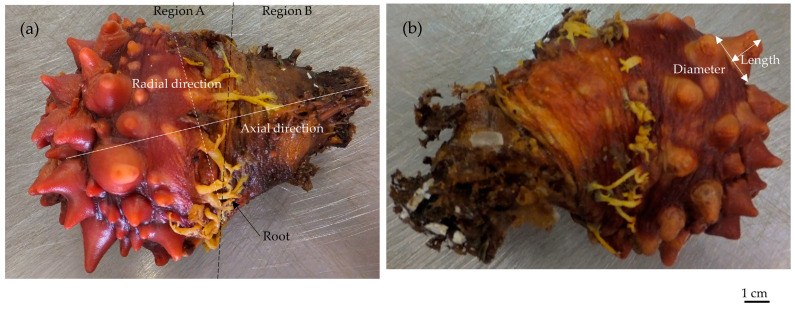
The sample of *Halocynthia roretzi*. (**a**) The tunic, covering the body, is divided into the two regions (Regions A and B) by the roots. The axial and radial directions are used to evaluate the tunic size. (**b**) The diameter and length represent the siphon size.

*Halocynthia roretzi* (Ascidiacea) (Figure 1) is covered with a tunic, the thickness of which is maintained through continual proliferation and removal [1]. Because of its physiological activities, this tunic is not only a cover for the entire body but also comprises tissues with active functions [1,2]. The components of this tunic have been extensively reported, including cellulose Iβ [3,4,5], sulfated chitin [6,7], pseudokeratin [8], α-smooth muscle actin [9], F-actin [9], and elastic fiber [9]. Active deformation in the tunic due to various stimuli, such as neurotransmitter (acetylcholine) [9], mechanical stimuli [9,10], electrical stimuli [11], and α-chymotrypsin [11], whose substrate is the same as that secreted by the hemocytes of *H. roretzi* [12]. The nervous system in the tunic has been observed [9,13], which manages the active deformation of the tunic. Also, various secretions by hemocytes have been reported [12,14,15,16,17,18,19,20], and thus, the condition of the tunic could be controlled by these hemocytes, which move around in the tunic because of its open circulatory system [21]. The tunic is composed of an outer region, a middle region, and an inner region: the outer region is the densest among these regions; the middle region is composed of laminar layers; and the inner region comprises loose layers [9]. Without the inner region and most of the middle region, the tunic could still respond to mechanical stimuli and actively deform [9]. During the active deformation of the tunic, changes in mass, which are largely influenced by the influx and efflux of water, have been reported [22]. While the shape of the tunic is complicated, as shown in Figure 1, changes in mass at the region close to the siphon (Region A in Figure 1) would be larger than those close to the bottom (Region B) [22]. The characteristics of the substances, which are related to the changes in mass, have also been examined [23]. While analyses to identify the components of the tunic and stimulants that trigger deformation have been carried out, the mechanical environment in the tunic, knowledge of which would be necessary for understanding the mechanism underlying deformation, has been barely investigated. Also, while the behaviors of the tunic related to deformation have been analyzed from various viewpoints [9,10,11,21,22,23], no system to integrate these findings has been proposed. Considering that the tunic has blood vessels and hemocytes on the inside and outside of the blood vessels because of its open circulatory system, the mechanical environment in the tunic could be properly controlled through blood circulation, where the behaviors of the hemocytes should not be prevented.

Cellulose, one of the most abundant resources in the world, has been examined in terms of various qualities, such as its crystalline [24] and mechanical properties [25] and its relation with water [26]. Thus, the versatile characteristics of cellulose have been successfully and widely applied [27]. Its electroactive characteristics have enabled the development of cellulose-based actuators [28,29,30,31,32,33,34]. In the meantime, chitin, which is another most abundant resource, has also shown usefulness in various fields as well as diverse characteristics [35,36,37,38,39], including sulfated chitin [35,38,39,40]. As previous reports have shown, sulfated polysaccharides have been observed in the tunic of ascidians [41,42,43,44,45,46,47,48,49,50]. Because sulfated polysaccharides are useful for biomedical applications, the methods of sulfation and desulfation of polysaccharides have been investigated [51]. However, the tunic of ascidians, which is composed of cellulose and sulfated chitin, has rarely been examined, except in *H. roretzi*. Also, recent progress in research on hydrogel [52] has introduced the proposals of cellulose hydrogel [53,54,55], chitin hydrogel [38,56,57,58,59], and chitin–cellulose hydrogel [60,61]. Despite the success of these proposals, sulfated chitin–cellulose hydrogel has not been developed. Moreover, due to the unique characteristics of ascidians, previous reports have indicated that vanadium in the hemocytes of ascidians has versatile roles, including as an inhibitor and an antioxidant [62,63,64,65,66].

Considering that neither an actuator nor a hydrogel that is composed of cellulose and sulfated chitin has been developed despite their useful characteristics, the tunic of *H. roretzi*, which is composed of both polysaccharides and shows active deformation, was analyzed in this study using the finite element method. Understanding the mechanical environment of its tunic, as evaluated by means of computation, will be helpful for designing actuators and hydrogels because this mechanical environment directly influences the deformation. The proposed model was based on the histological and mechanical characteristics related to active deformation reported in previous studies.

## 2. Materials and Methods

**Figure 2:**
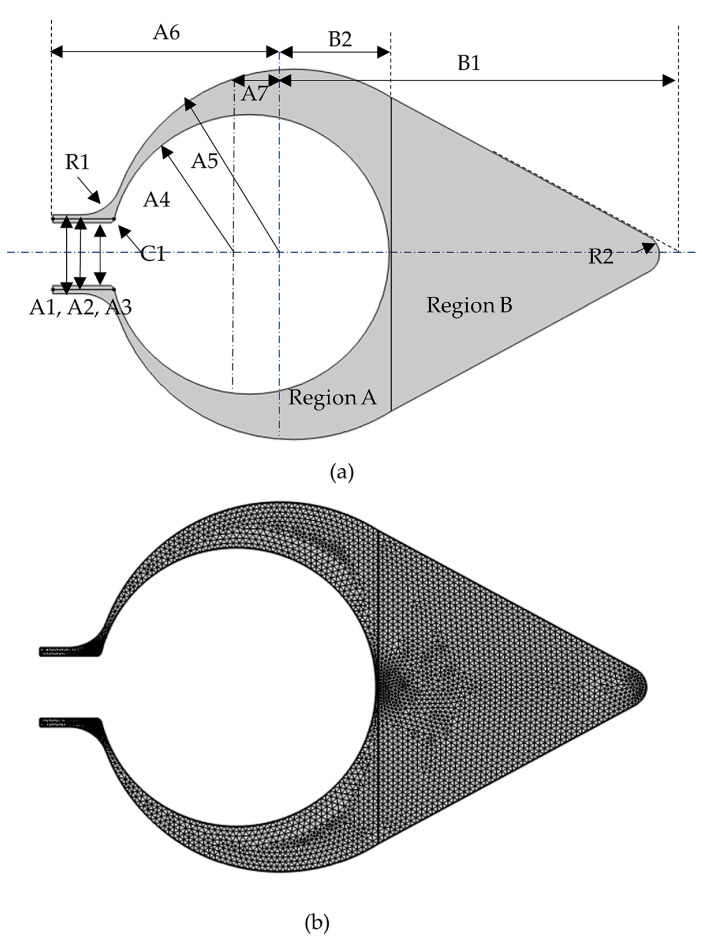
Two-dimensional finite element model for the tunic of *H. roretzi*. This model corresponds to the cross section of the tunic: the right side is the siphon and the left side is the bottom. (**a**) is the entire image of the model, whose shape is determined by the parameters (A1–A7,B1,B2,C1,R1,R2), as shown in Table 1. The boundary between Regions A and B corresponds to the region of the roots. (**b**) shows 6243 triangular elements in the model.

**Figure 3:**
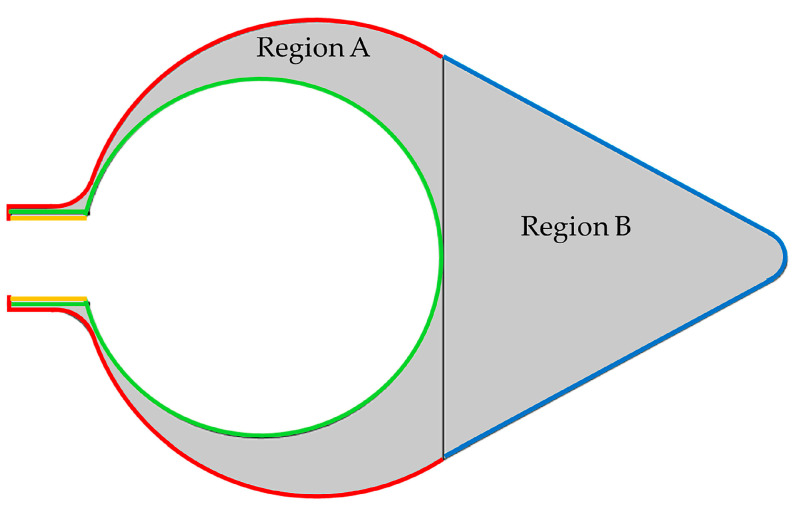
Components in the finite element model of the tunic: there are two regions (Regions A and B) and three layers (LA-1 (Red), LA-2 (Green), LA-3 (Orange), and LB (Blue)). The mechanical properties are shown in Table 2.

All computations were carried out using a software for finite element analysis (COMSOL Multiphysics^®^, Version 6.1). Figure 1 shows the samples, which were used for size measurements to develop the model. Regions A and B contact each other at the region of the roots. The tunic model proposed in this study is shown in Figure 2, and the sizes of the model are indicated in Table 1 and described in Section 2.1. The model corresponds to the cross section of *H. roretzi* without its muscle and internal organs. Additionally, the model has one siphon despite the presence of two siphons because their relative configuration was difficult to determine, and this study focused on the relationship between the main body and siphon. This model is fixed at the curve pointed out by the arrow, B2. The layers, which were set around Regions A and B in the model, are shown in Figure 3. Table 2 and Table 3 show the mechanical properties and load in each region, respectively. The following sections provide a description on how to set the sizes and mechanical properties of the components and the load.

### 2.1. Size

Table 1 shows the sizes used in the proposed model. Figure 1 shows the sample of *H. roretzi* (Marutaki Suisan, Miyagi, Japan). To evaluate the size of the tunic, the distances along the radial and axial directions were measured (Figure 1a): the former corresponds to the largest distance without projections, and the latter is the distance from the bottom to the region just below the siphon. The size of the siphon, including diameter and length, is shown in Figure 1b.

As shown in Figure 2a, this model is composed of the cross section of the siphon (two rectangles), Region A except the siphon (two circles) and Region B (rectangle, one of its vertices is replaced with an arc (R2 in Figure 2)). Each region is connected smoothly so that the boundary between the siphon region and Region A is connected with a curve (R1). The vertex at the inside of the siphon is cut off (C1). The starting and end points of A2 in Figure 2a correspond to the region where the muscle lies between the folded tunic at the top of the siphon. The thickness of the tunic is dependent on its position: it is smallest around the siphon and thickest around the bottom based on the sample observation. The inner surface, which contacts the muscle, passes around the inside of the root region. Hence, the inner surface of the tunic is approximated as a circle, whose closest distance from the boundary between Regions A and B is 0.51 mm. In the meantime, the tunic can be categorized into the following three regions based on the distribution pattern of the filaments: the outer region, which is densest; the middle region, which is laminar; and the inner region, which is loose [9]. Because it is difficult to evaluate the thickness of each layer, the outer and inner layers are set as a thin layer (nonlayered). The thickness of the outer layer at the bottom is twice as large as those at other regions (Table 2): the difference in the layer thickness is about 1% of the difference between the centers of the inner and outer surfaces, A7, as shown in Figure 2 and Table 1.

### 2.2. Mechanical Properties and Load

The mechanical properties and load are shown in Table 2 and Table 3, respectively. Deformation is accompanied by changes in mass, which is more evident at the region closer to the siphon [22], so that Regions A and B are compressible and incompressible in the model, respectively. The outer and inner layers were set as compressible. The Poisson’s ratio in the compressible part was set to be 0.3 [67], while that in the incompressible part was set to be 0.4999. Because mechanical stimuli induce active deformation of the tunic [9,10,22], the elastic modulus of the tunic is hard to measure. Considering that the tunic region closer to the siphon is more deformable than that closer to the bottom [9,22], the elastic modulus will increase when the position is closer to the bottom. Hence, the elastic modulus of Region B (1 × 10^4^ Pa) was set to be larger than that of Region A (5 × 10^3^ Pa). These elastic moduli were thought to be similar to those in the middle region, the laminar layer [9], at Regions A and B, respectively. The fiber distribution in the outer and inner regions is dense and loose, respectively [9]; thus, the elastic modulus in each region was set as larger and smaller than each region: LA-1 (the outer region in Region A) was set to be 5 × 10^4^ Pa; LA-2 (the inner region in Region A) was set to be 1 × 10^3^ Pa; LA-3 (the outer region of the siphon, similar to the inner region [22]) was set to be 1 × 10^3^ Pa; and LB (the outer region in Region B) was set to be 1 × 10^5^ Pa. Comparing these elastic moduli with that of whisker crystalline cellulose Iβ, which is 143 GPa [25], the elastic moduli in this model are less than 1% of that in whisker crystalline. The tunic deforms inside when it is cut. The deformation occurs because residual stress is released via the cut. Hence, the inner and outer regions are stretched and compressed under physiological conditions, respectively. In terms of residual stress, just a small amount was set for the inner and outer layers, as shown in Table 2.

When the middle and inner regions of the tunic are removed, the outer region of the tunic actively deforms [9]. Supposing that the outer region controls the deformation, the thickness of the outer region could be a parameter for the load. When the body is enlarged, the outer and inner regions need to be compressed and stretched, respectively. Hence, the load at each layer was set as follows (Table 3): the outer region (compressive load), LA-1 (5 N/m) and LA-3 (0.5 N/m) (Region A), LB (10 N/m) (Region B), the inner region (tensile load), and LA-2 (0.01 N/m) (Region A). The developed model is named “Control”.

### 2.3. Influence of Mechanical Parameters

To evaluate the influence of residual stress on stress distribution in the tunic, a model without residual stress and with the same load (No-RS) was developed. Tunic deformation with the influx and efflux of water is larger when the tunic region is closer to the siphon [22]. Hence, Regions A and B are, respectively, compressible and incompressible in the Control model. To evaluate the influence of compressibility on the mechanical environment in the tunic, two models were developed: ICOM, in which all regions and layers are incompressible, and COM, in which all regions and layers are compressible.

### 2.4. Parameters for the Mechanical Environment

**Figure 4:**
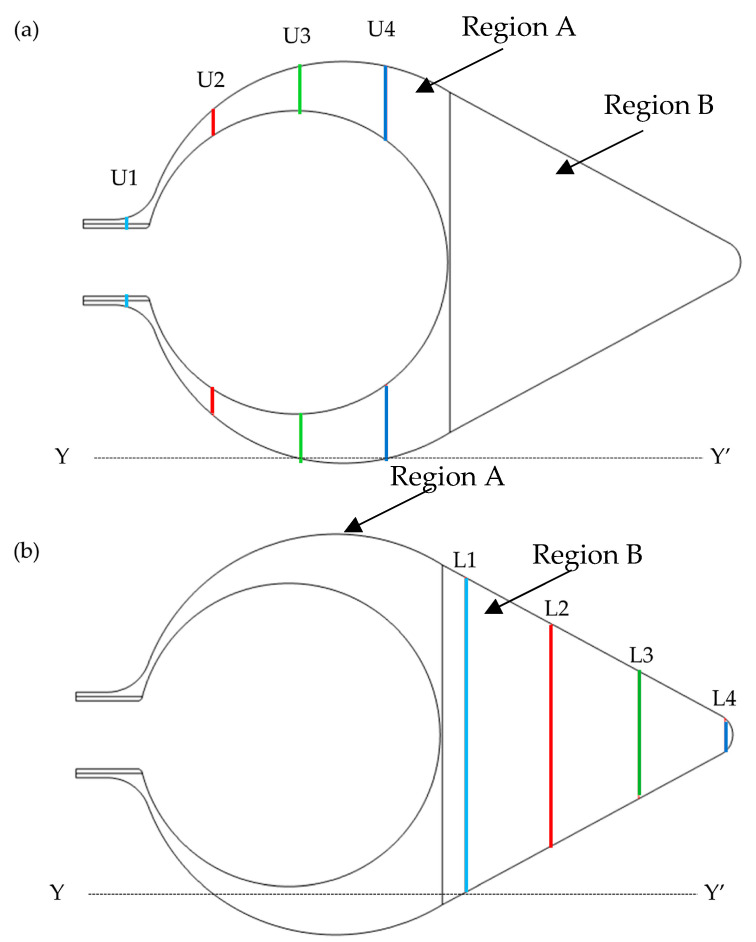
Evaluation of the mechanical environment in the tunic. Each mechanical parameter was evaluated along the lines in each region: (**a**) U1, U2, U3, and U4 in Region A, and (**b**) L1, L2, L3, and L4 in Region B. Lines are parallel to each other at every 20 mm.

The following parameters were used to evaluate the mechanical environment in the tunic: von Mises stress, displacement, and pressure. The distribution pattern of each parameter was visualized in the entire tunic. Also, the values of each parameter along the lines, which were set in the model as shown in Figure 4, were plotted as graphs.

## 3. Results

The three parameters (von Mises stress, pressure, and displacement) in the four models (Control, No-RS, ICOM, and COM) are described in the following sections.

### 3.1. von Mises Stress

**Figure 5:**
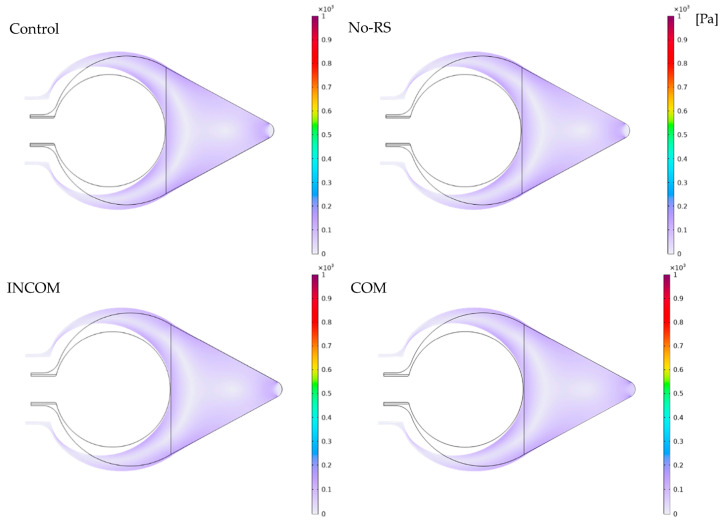
von Mises stress in the tunic when the body is enlarged (Regions A and B). Control, initial condition; No-RS, no residual stress; INCOM, incompressible in all components; COM, compressible in all the component.

**Figure 6:**
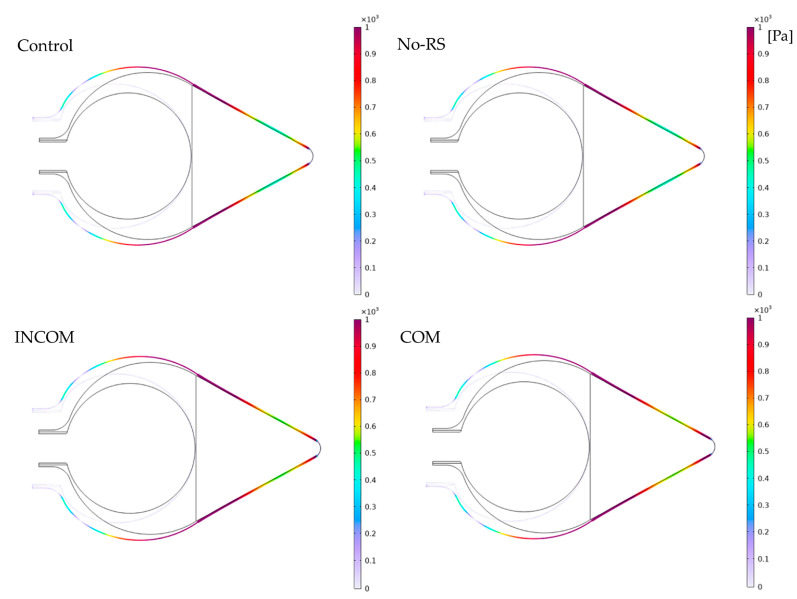
von Mises stress in the tunic when the body is enlarged (all the layers). Control, initial condition; No-RS, no residual stress; INCOM, incompressible in all components; COM, compressible in all components.

**Figure 7:**
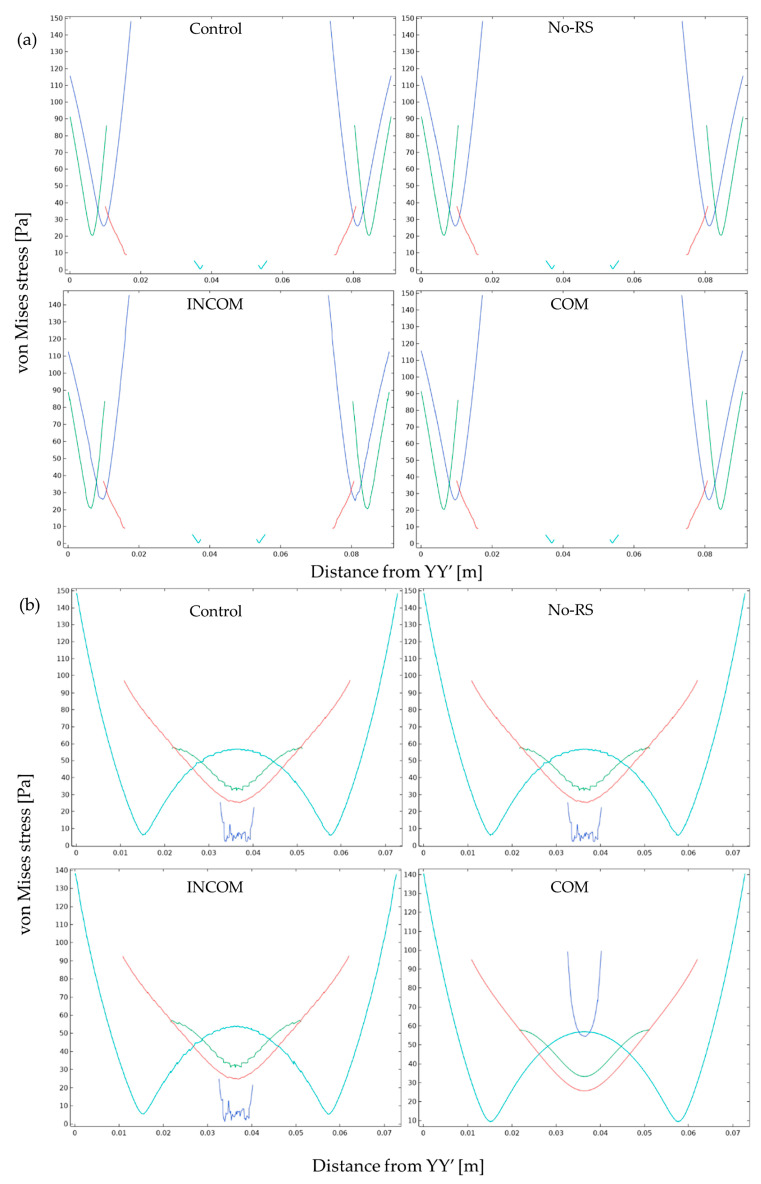
von Mises stress in the tunic when the body is enlarged. von Mises stress along the line indicated in Figure 4 is shown. The color in each line was corresponding to that in Figure 4. (**a**), Region A; (**b**), Region B. Control, initial condition; No-RS, no residual stress; INCOM, incompressible in all components; COM, compressible in all components.

Figure 5 shows the distribution of von Mises stress in Regions A and B in the four models. The distribution of von Mises stress was almost uniform in all models, except the regions around the boundary between Regions A and B, and the fixed region at the bottom, where the stress was close to zero. Figure 6 shows von Mises stress along all layers (LA-1, LA-2, LA-3, and LB). At the boundary between Regions A and B and the fixed region at the bottom, von Mises stress was much higher than in the other regions for all models. The von Mises stress along the lines, as depicted in Figure 4, is shown in Figure 7. In Region A, the distribution pattern along the line was almost the same in each model. However, the result of INCOM indicated the value was lower than those in the other models. Hence, increased incompressibility of the tunic decreases von Mises stress. In Region B, the results of the three models, Control, No-RS, and ICOM, where Region B was incompressible, indicated fluctuations, while the distribution of COM was smooth. Also, around the fixed region at the bottom, COM showed the highest stress among all models. Control and No-RS showed stresses that were higher than those of INCOM and COM at the boundary between Regions A and B. Compressibility suddenly changed in Control and No-RS, while such a change did not occur in INCOM and COM. This change would cause an increase in von Mises stress in Control and No-RS.

### 3.2. Pressure

**Figure 8:**
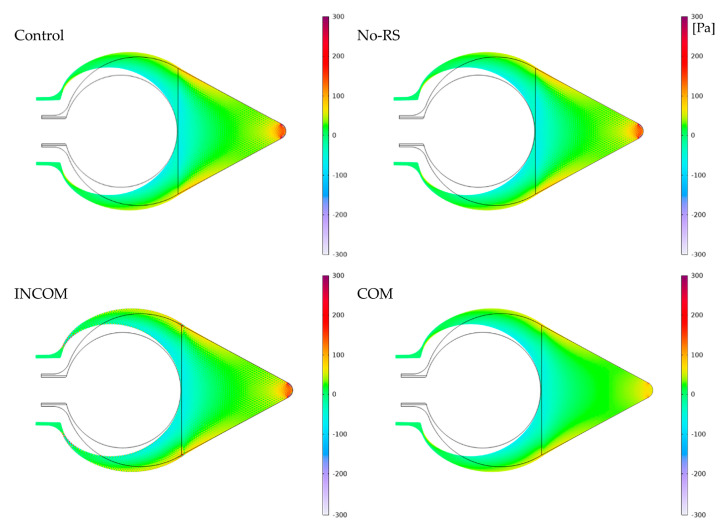
Pressure in the tunic when the body is enlarged (Regions A and B). Control, initial condition; No-RS, no residual stress; INCOM, incompressible in all components; COM, compressible in all components.

**Figure 9:**
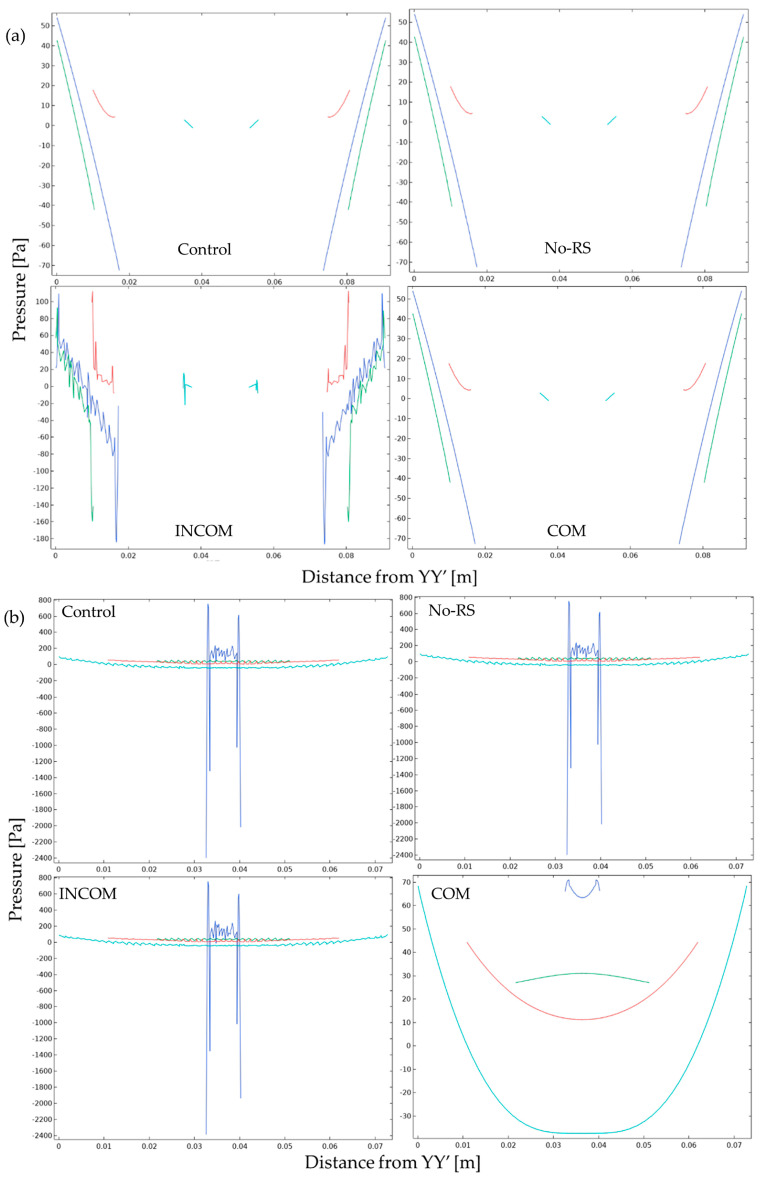
Pressure in the tunic when the body is enlarged. Pressure along the line indicated in Figure 4 is shown. The color in each line was corresponding to that in Figure 4. (**a**), Region A; (**b**), Region B. Control, initial condition; No-RS, no residual stress; INCOM, incompressible in all components; COM, compressible in all components.

**Figure 10:**
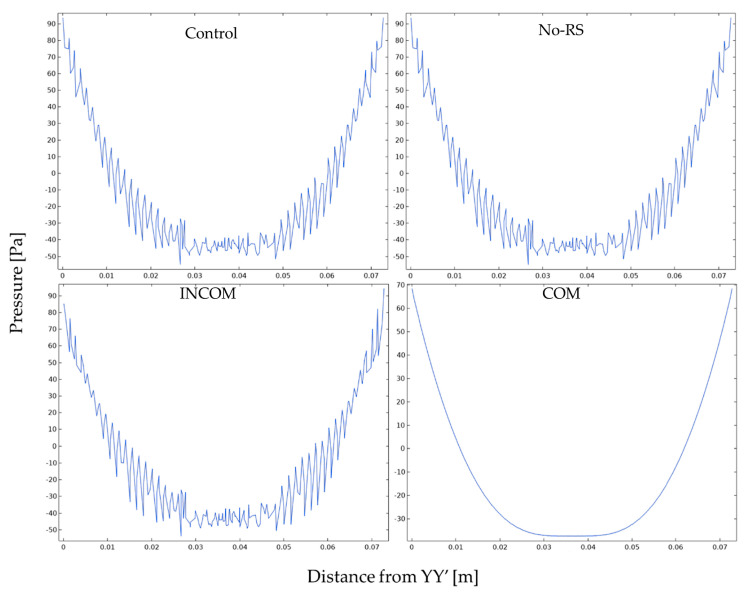
Pressure in the tunic along the line L4 in Region B. Control, initial condition; No-RS, no residual stress; INCOM, incompressible in all components; COM, compressible in all components.

Figure 8 shows the distribution of pressure in Regions A and B in the four models. Region A showed a lower pressure than Region B in all models. However, the pressure around the fixed region at the bottom in COM was much lower than that of the other models. The pressure along the lines, as indicated in Figure 4, is shown in Figure 9. In Region A, all models showed almost the same pattern of pressure. However, the pressure in ICOM fluctuated and was higher than that in the other models. In Region B, control, No-RS, and ICOM showed fluctuated pressure, while pressure in COM was distributed smoothly. Figure 10 shows the pressure along L4, the line closest to the boundary in Region B; the pressure along L4 seems smooth in all models, as shown in Figure 9. The pressure in COM was distributed smoothly, while that in the other models largely fluctuated.

### 3.3. Displacement

**Figure 11:**
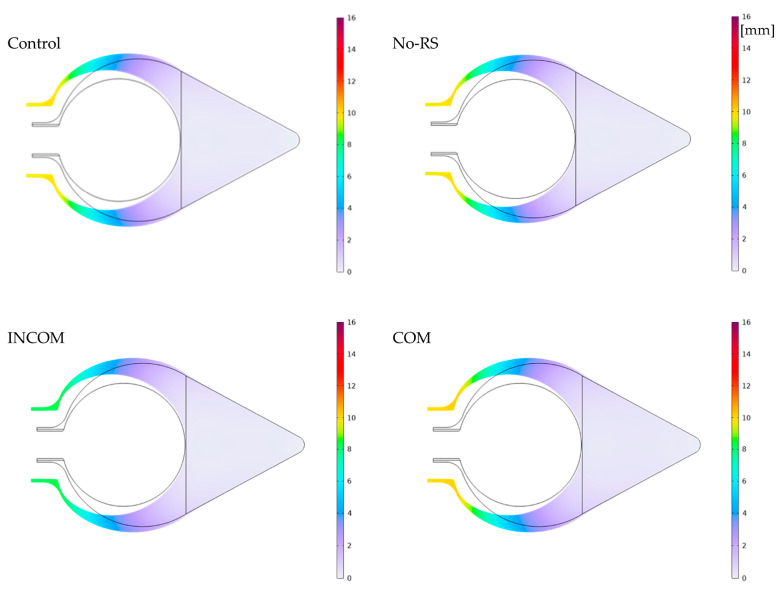
Displacement of the tunic when the body is enlarged (Regions A and B). Control, initial condition; No-RS, no residual stress; INCOM, incompressible in all components; COM, compressible in all components.

**Figure 12:**
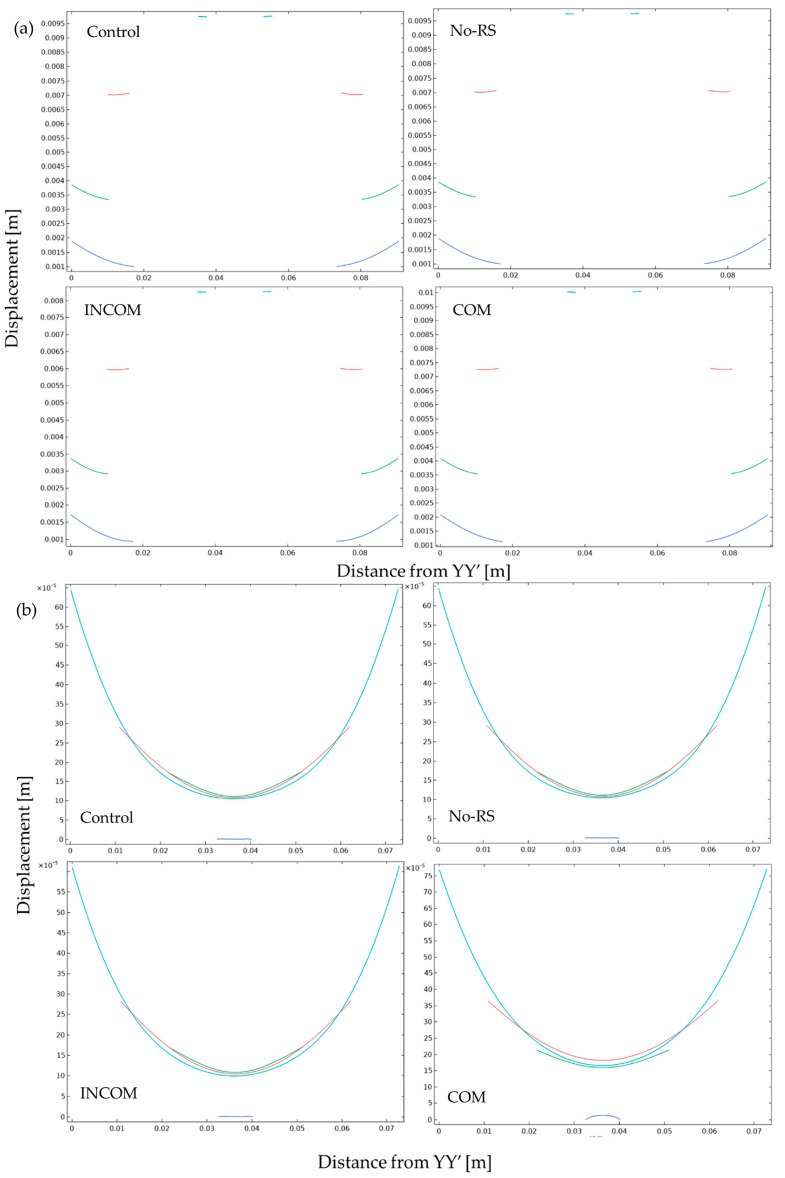
Displacement of the tunic when the body is enlarged. Displacement along the line indicated in Figure 4 is shown. The color in each line was corresponding to that in Figure 4. (**a**), Region A; (**b**), Region B. Control, initial condition; No-RS, no residual stress; INCOM, incompressible in all components; COM, compressible in all components.

Figure 11 shows the distribution of displacement in Regions A and B. In all models, displacement in the siphon region became higher than in the other regions. The siphon region in ICOM showed lower displacement than that in the other models. At the boundary between Regions A and B, where blood vessels are observed, the displacement became lower than in the other regions for all models. Figure 12 shows the displacement along the lines indicated in Figure 4. In Regions A and B, the displacement in INCOM was most reduced among all models, while that in COM was most enhanced.

## 4. Discussion

Cellulose and chitin are versatile as well as abundant; thus, their effective usage will influence a large number of research fields. While research fields related to these polysaccharides, including actuators, have made progress, actuators based on cellulose and sulfated chitin have barely been developed. Meanwhile, previous reports have shown that the tunic of *H. roretzi* is composed of cellulose and sulfated chitin, and it actively deforms with the influx and efflux of water. Even though the mechanical environment in the tunic could directly influence deformation, limited investigation has been carried out. Also, understanding the characteristics of the mechanical environment could be helpful for designing actuators. Even though the tunic of *H. roretzi* responds to mechanical stimuli, its mechanical parameters have rarely been measured. Hence, the finite element method was used for this investigation. In this study, a finite element model for the tunic of *H. roretzi* was proposed in order to evaluate its mechanical environment.

This model was developed based on the deformation patterns and histological characteristics indicated in previous reports as follows:

- The region close to the siphon deforms more than that close to the bottom. Hence, the elastic modulus at the region close to the siphon is smaller than that close to the bottom in this model.
- Deformation is accompanied by the influx and efflux of water. Hence, the region close to the siphon is compressible, while that close to the bottom is incompressible in this model.
- The tunic deforms on the inside after cutting so that there is residual stress. Hence, residual stress was set for this model.
- The outer region of the tunic could cause deformation. Hence, the main load was set to exert on the outer region in this model.
- The tunic could be categorized into three regions based on fiber distribution: the outer region is dense; the middle region is laminar; and the inner region is loose. Hence, the magnitude of the elastic modulus in each region is as follows: the outer region > the middle region > the inner region.

In this study, the influence of compressibility and residual stress was evaluated. All mechanical parameters showed smaller values at the boundary between Regions A and B, where blood vessels are often observed. Because all models showed the same tendency, the results could be influenced by the characteristics of the shape of the tunic. Also, the parameters were reduced and fluctuated with increased incompressibility. On the contrary, compressibility caused smooth deformation. The effect of residual stress was hardly observed. At the outer layers, von Mises stress was much larger than in Regions A and B, at the boundary for these regions and around the fixed region at the bottom. Compressibility of the tunic, which is directly related to the influx and efflux of water in the tunic, could influence the mechanical environment of the tunic. Considering that sulfated chitin is hydrophilic, it would be useful for the control of water content in the tunic. As shown by the results, the outer region of the tunic showed higher von Mises stress than the inside; thus, the pattern of fiber distribution could help maintain the mechanical environment in the tunic. Hence, the shape of the tunic, the pattern of fiber distribution in the tunic, and the influx and efflux of water influence the mechanical environment in the tunic. The geometrical effect, pattern of fiber distribution in space, and control of water content will be helpful when designing a cellulose–sulfated chitin actuator. In this study, all mechanical parameters were estimated based on the histological characteristics. Hence, an experimental method to properly evaluate the mechanical parameters of the tunic will be developed in the future.

## 5. Conclusions

The mechanical environment in the tunic of *H. roretzi*, which is a cellulose–sulfated chitin tissue with active deformation, was evaluated using the finite element method. The geometrical aspect, fiber distribution patterns, and control of the water content influenced the mechanical environment in the tunic. These results would be helpful for designing a cellulose–sulfated chitin actuator.

## Figures and Tables

**Table 1:** Sizes set in the computational model of the tunic.

Parameter *	Size [mm] **
A1	19.75
A2	17.75
A3	15.75
A4	35
A5	46.36
A6	60.04
A7	10.85
B1	97.29
B2	24.66
R1 ***	0.5
R2 ***	1
C1 ****	0.5

*, all parameters are shown in Figure 2. **, all sizes were obtained from two samples, as described in Section 2.2. ***, the radius of the curvature. ****, the distance from the vertex for cutting off.

**Table 2:** Mechanical properties of the computational model of the tunic.

Component	Elastic Modulus [Pa]	Poisson’s Ratio	Thickness [mm]	Residual Stress [Pa]	
				Magnitude	Type
Region A	5 × 10^3^	0.3	-	-	
Region B	1 × 10^4^	0.4999	-	-	
LA-1	5 × 10^4^	0.3	0.1	0.01	Compressive
LA-2	1 × 10^3^	0.3	0.1	0.01	Tensile
LA-3	1 × 10^3^	0.3	0.1	0.01	Compressive
LB	1 × 10^5^	0.3	0.2	0.1	Compressive

**Table 3:** Load on the computational model of the tunic.

Component		Load [N/m]	
Category	Type	Magnitude	Type
LA-1	Outer region (Main (except siphon))	5	Compressive
LA-3	Outer region (siphon)	0.5	Compressive
LB	Outer region (bottom)	10	Compressive
LA-2	Inner region	0.01	Tensile

## Data Availability

The data presented in this study are available from the corresponding author upon request.
